# Complete chloroplast genome sequence of the red silk cotton tree (*Bombax ceiba*)

**DOI:** 10.1080/23802359.2017.1422399

**Published:** 2018-03-05

**Authors:** Yong Gao, Haibo Wang, Chao Liu, Honglong Chu, Yuehui Yan, Lizhou Tang

**Affiliations:** a College of Biological Resource and Food Engineering, Center for Yunnan Plateau Biological Resources Protection and Utilization, Qujing Normal University, Qujing, China;; b Key Laboratory of Yunnan Province Universities of the Diversity and Ecological Adaptive Evolution for Animals and Plants on YunGui Plateau, Qujing Normal University, Qujing, China

**Keywords:** *Bombax ceiba*; complete chloroplast genome; Bombacaceae

## Abstract

*Bombax ceiba* L. is a beautiful and deciduous tree with great ecological and economic importance. The third generation sequencing of chloroplast genome of *B. ceiba* was conducted on the PacBio sequencing platform (Pacific Biosciences). The complete chloroplast genome was 158,997 bp, which contains a large single-copy (LSC) region (89,021 bp), a small single-copy (SSC) region (21,110 bp), and two inverted repeats (IRs) (24,433 bp). In total, 116 genes were annotated, including 81 protein-coding genes, eight rRNA genes, and 27 tRNA genes. The phylogenetic tree showed that *B. ceiba* was closely clustered with one clade of Malvaceae.

*Bombax ceiba* L., commonly known as red silk cotton tree, belongs to the family Bombacaceae. It is a beautiful and deciduous tree with red and large flowers. This species is widely distributed in tropical and sub-tropical Asia, Africa, and Australia (Jain et al. 2009). *B. ceiba* has specific role in economy for its cotton and timber in many Asian countries. Many parts of this plant are used by various tribal communities for the treatment of a variety of ailments (Pankaj and Somshekhar 2012). *B. ceiba* is also regarded as the city flower of Guangzhou city in China for its vivid and beautiful flowers. In this study, we characterized the intact chloroplast genome of *B. ceiba* to help with the further molecular and phylogenetic studies of this plant.

The leaf sample of *B. ceiba* was collected from Yuanmou, Yunnan Province, China (25°40′50.06″ N, 101°53′27.76″ E). Genomic DNA was extracted using the DNeasy Plant Mini Kit (QIAGEN, Hilden, Germany). The genomic DNA and surplus leaf materials were stored in a −80 °C refrigerator in the key laboratory of Yunnan province universities of the diversity and ecological adaptive evolution for animals and plants on YunGui plateau, Qujing Normal University. PacBio SMRTbell DNA library preparation and sequencing (P6, C4 chemistry) were performed by NextOmics (Wuhan, China) according to the protocols (Pacific Biosciences, Menlo Park, CA). With chloroplast genome sequences of 26 other Malvaceae species as the reference genomes, the PacBio long reads of cpDNA were filtered from the whole genome sequences of *B. ceiba*. A *de novo* assembly of the chloroplast genome of *B. ceiba* was performed using CANU software (Koren et al. 2017). The complete chloroplast genome was first annotated by CpGAVAS (Liu et al. 2012), and then manually adjusted.

The chloroplast genome of *B. ceiba* (GenBank accession MG569974) was 158,997 bp in length, with a GC content of 36.81%. The chloroplast genome was comprised of a large single-copy (LSC) region (89,021 bp), a small single-copy (SSC) region (21,110 bp), and two inverted repeats (IRs) (24,433 bp). It contained 116 genes, including 81 protein-coding genes, 27 tRNA genes, and eight rRNA genes. For the two IR regions, 15 genes were duplicated including eight protein-coding genes, three tRNAs, and four rRNAs.

To validate the phylogenetic position of *B. ceiba*, complete cpDNA sequences of 11 Malvales species and four other plants were downloaded from the National Center for Biotechnology Information (NCBI) database. The sequences were first aligned by MUSCLE v3.8.31 (Edgar 2004). Then, a maximum-likelihood phylogenetic tree was constructed using Mega v7.0 (Kumar et al. 2016) with the generalized time-reversible (GTR) model and 1000 bootstraps. The phylogenetic tree suggested that *B. ceiba* was clustered with one clade of Malvaceae (*Hibiscus hamabo*, *Hibiscus syriacus*, and *Abelmoschus esculentus*) (Figure 1), which was different from the previous study (Baum et al. 2004). In conclusion, the complete cpDNA sequence of *B. ceiba* is reported in this study. It provides additional genomic resources for further phylogenetic and evolutionary analysis of *B. ceiba*.

**Figure 1.**
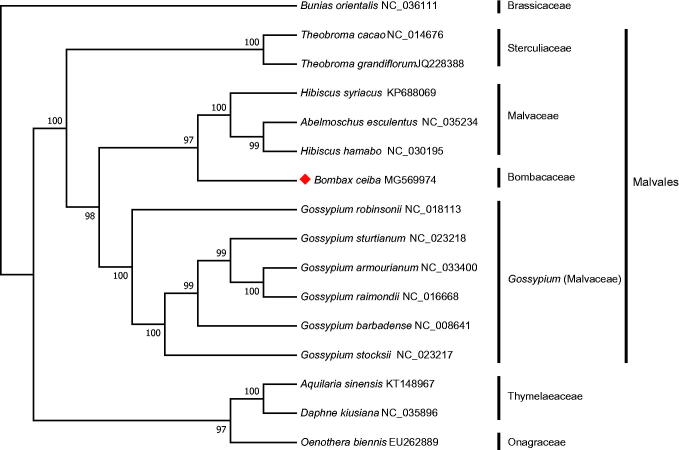
The maximum-likelihood phylogenetic tree constructed with chloroplast genomes of 16 plants. Bootstrap values are shown at the nodes. The chloroplast genome accession number used in the phylogeny study: *Bunias orientalis*: NC_036111; *Theobroma cacao*: NC_014676; *Theobroma grandiflorum*: JQ228388; *Hibiscus syriacus*: KP688069; *Abelmoschus esculentus*: NC_035234; *Hibiscus hamabo*: NC_030195; *Bombax ceiba*: MG569974; *Gossypium robinsonii*: NC_018113; *Gossypium sturtianum*: NC_023218; *Gossypium armourianum*: NC_033400; *Gossypium raimondii*: NC_016668; *Gossypium barbadense*: NC_008641; *Gossypium stocksii*: NC_023217; *Aquilaria sinensis*: KT148967; *Daphne kiusiana*: NC_035896; *Oenothera biennis*: EU262889.
